# Clinical benefit of subsequent chemotherapy after drug-induced interstitial lung disease in pancreatic cancer patients: a multicenter retrospective study from Japan

**DOI:** 10.1186/s12885-023-10781-x

**Published:** 2023-04-06

**Authors:** Hiroki Irie, Rei Suzuki, Yoshinori Okubo, Hiroyuki Asama, Naoki Konno, Yuki Noguchi, Ko Watanabe, Goro Shibukawa, Hidemichi Imamura, Tadayuki Takagi, Mitsuru Sugimoto, Yuki Sato, Jun Nakamura, Tsunetaka Kato, Minami Hashimoto, Takumi Yanagita, Takuto Hikichi, Hiromasa Ohira

**Affiliations:** 1 Department of Gastroenterology, Fukushima Medical University School of Medicine, 1 Hikarigaoka, Fukushima, 960-1295 Japan; 2 Department of Gastroenterology, Fukushima Rosai Hospital, Fukushima, Japan; 3 Department of Gastroenterology, Fukushima Red Cross Hospital, Fukushima, Japan; 4 Department of Gastroenterology, Ohta Nishinouchi Hospital, Fukushima, Japan; 5 Department of Gastroenterology, Ohara General Hospital, Fukushima, Japan; 6 Department of Gastroenterology, Fukushima Medical University Aizu Medical Center, Fukushima, Japan; 7 Department of Endoscopy, Fukushima Medical University Hospital, Fukushima, Japan

**Keywords:** Pancreatic ductal adenocarcinoma, Chemotherapy, Gemcitabine, Nab-paclitaxel, Interstitial lung disease

## Abstract

**Purpose:**

Drug-induced interstitial lung disease (ILD) is not a rare adverse event in the current chemotherapy strategy for pancreatic ductal adenocarcinoma (PDAC). Thus, we aimed to find the optimal management for PDAC patients with a history of ILD induced by a gemcitabine-based regimen.

**Methods:**

We conducted a multicenter retrospective study. The primary endpoint was the overall survival (OS) of patients who underwent either S-1 monotherapy or FOLFOX after the onset of ILD. Toxicity data was also analyzed in the 2 groups.

**Results:**

Twenty-four patients were diagnosed with ILD and 17 patients who received subsequent chemotherapy were enrolled in the study. Among 17 patients who were managed with subsequent chemotherapy after recovering from ILD, we did not observe significant difference in OS between S-1 and FOLFOX (290.0 days vs. undefined, p = 0.39). Relapse of drug-induced ILD was not observed in all cases during the course. Overall, severe adverse events (CTCAE Grade 3 or 4) were observed in 3 patients (23.1%) in S-1 treatment group and 1 patient (25.0%) in FOLFOX treatment group (p = 0.93).

**Conclusions:**

S-1 monotherapy and FOLFOX are comparable as the subsequent chemotherapy after gemcitabine-based chemotherapy-induced ILD in unresectable PDAC.

## Introduction

Pancreatic ductal adenocarcinoma (PDAC) is one of the most lethal malignancies in the world [1, 2]. Currently, there are two first-line chemotherapy options for PDAC patients. Compared with gemcitabine treatment, FOLFIRINOX (FFX) triplet therapy (fluoropyrimidin, oxaliplatin and irinotecan) is associated with improved overall survival (OS) and is the standard first-line chemotherapy for fit patients with metastatic PDAC [3, 4]. Another regimen, the combination of gemcitabine and nab-paclitaxel (GnP), is also associated with improved OS compared with gemcitabine alone [5, 6]. While subsequent studies have not found a significant difference in treatment efficacy between the two regimens, GnP is preferably used in clinical practice due to its relatively mild toxicity [7–10].

As mentioned above, gemcitabine-based chemotherapy is indispensable in the treatment of unresectable PDAC; however, real-world studies have raised concerns that these treatments may induce more adverse events, including interstitial lung disease (ILD), than those reported in clinical trials. In the MPACT trial, the incidence of ILD was reported to be less than 1% while the specific etiology was not described [5]. On the other hand, recent real-world studies have shown that ILD might occur more often with gemcitabine-based regimens, and the incidence has been reported to be up to 20% (2.2–20%) [11–15]. Currently, there are no data available regarding whether PDAC patients after recovering from ILD should be managed with or without subsequent chemotherapy. Permanent discontinuation of antineoplastic agents is encouraged in certain situations, while readministration of those agents has been shown to improve patient prognosis in other situations [16]. Regarding subsequent chemotherapy, it has not been evaluated which regimen should be selected. The present study aimed to find the optimal management for PDAC patients with a history of ILD induced by a gemcitabine-based regimen.

## Materials and methods

### Study design

This multicenter retrospective study evaluated data from consecutive patients who underwent palliative chemotherapy after experiencing ILD induced by a gemcitabine-based regimen for advanced PDAC at Fukushima Medical University Hospital and 5 related facilities (Fukushima Medical University Aizu Medical Center, Fukushima Rosai Hospital, Ohara General Hospital, Fukushima Red Cross Hospital and Ohta Nishinouchi Hospital) between December 2014 and June 2022. All patients were pathologically diagnosed with PDAC, and patients with rare primary pancreatic neoplasms, including acinar cell carcinoma or neuroendocrine carcinoma, were excluded. The study protocol conformed to the ethical guidelines of the 1975 Declaration of Helsinki and was approved by the institutional ethical committee of Fukushima Medical University (Fukushima, Japan; IRB number #29,254). The need for informed consent was waived because of the retrospective design.

### Treatment

After recovering from ILD, the decision was made by each patient’s physician whether the patient would be managed with or without subsequent chemotherapy (S-1 monotherapy or FOLFOX). We did not choice irinotecan and nano-liposomal irinotecan since those agents were contraindicated for patients with a history of ILD. Patients allocated to S-1 alone received S-1 orally twice daily at a dose according to their body surface area (BSA) (< 1.25 m^2^, 80 mg/day; ≥ 1.25 to < 1.5 m^2^, 100 mg/day; ≥ 1.5 m^2^, 120 mg/day) on days 1 through 28 of a 42-day cycle [17]. As a modified regimen of FOLFINOX, the FOLFOX regimen was selected for some patients (oxaliplatin 85 mg/m2 in a 2-hour infusion, folinic acid 400 mg/m^2^ in a 2-hour infusion, followed by 5-FU bolus 400 mg/m^2^, then by 5-FU continuous infusion 2400 mg/m^2^ in 46 h) [18]. The toxicities were graded according to the National Cancer Institute Common Terminology Criteria for Adverse Events (CTCAE) version 5.0. A follow-up CT scan was obtained every 2 cycles of chemotherapy to evaluate the treatment effect unless abnormalities were found on the physical exam or in the laboratory data. All patients who received at least one cycle of posttreatment were included in the analysis.

### Subjects for analysis

#### Variables

Clinical characteristics before initiation of post-ILD chemotherapy (including age, sex, tumor stage, Eastern Cooperative Oncology Group performance status [PS], smoking history, Brinkman index [BI], history of lung disease, presence of emphysema on computed tomography [CT] scan and timing of gemcitabine-based treatment [1st line or later]) and levels of serum markers (carcinoembryonic antigen [CEA], cancer antigen 19 − 9 [CA19-9], Krebs von den Lungen-6 antigen [KL-6], surfactant protein D [SP-D]) at the onset of ILD were collected. Lung disease was defined as clinically diagnosed preexisting ILD, chronic obstructive pulmonary disease (COPD), bronchiectasis and asbestosis [19]. The severity of ILD was graded in accordance with the National Cancer Institute Common Terminology Criteria for Adverse Events (CTCAE) version 5.0. CTCAE grades were collected for pneumonitis. The primary endpoint was OS of the patients after onset of ILD. OS was calculated from the date of initiation of the treatment to the date of death or the last follow-up.

#### Statistics

Continuous variables are reported as the median (range). For categorical data, the chi-square test or Fisher’s exact test was performed, as appropriate. Continuous variables were compared using either the Mann–Whitney test or Kruskal–Wallis test. The median OS after initial chemotherapy was calculated using the Kaplan‒Meier method. All statistical analyses were performed with GraphPad Prism 9.0 (GraphPad, San Diego, CA, USA). p < 0.05 was considered statistically significant.

## Results

### Patient characteristics

We recruited 480 patients from 6 medical facilities, and 24 patients were diagnosed with ILD during gemcitabine-based chemotherapy. Among them, 17 patients who underwent subsequent chemotherapy were enrolled in the analysis. The clinical backgrounds of the selected patients are summarized in Table 1. Briefly, their median age was 71 years, 76.4% were male and 52.9% of the selected patients had a smoking history. Grade ≥ 2 ILD was confirmed in 10 patients (58.8%). Offending agents were discontinued in all cases, and short course steroid pulse therapy and subsequent oral steroids were administered in 5 patients with relatively severe disease. All patients recovered to Grade ≤ 1 before undergoing subsequent chemotherapy. The median recovery time between the onset of ILD and readministration of chemotherapy was 42 days (range: 6–76).

**Table 1:** Comparison of patients’ characteristics

	With subsequent chemotherapy n = 17	
**Age (year-old)**	**71 (45–76)**	
**Sex (male: female)**	**13:4**	
**Tumor stage IV, n (%)**	**11 (64.7)**	
**ECOG PS (0:1)**	**17:0**	
**Smoking history, n (%)**	**9 (52.9)**	
**Brinkman index**	**500.0 (0-1530)**	
**History of lung disease, n (%)**	**0 (0)**	
**Emphysema on CT scan, n (%)**	**4 (23.5)**	
**Treatment, 1st line vs. later**	**15 (88.2)**	
**CEA (ng/mL)**	**5.9 (1.3-109.1)**	
**CA19-9 (U/mL)**	**200.4 (2.1–8543.0)**	
**KL-6 (U/mL)**	**356.0 (187.0-1784.0)**	
**SP-D (ng/mL)**	**198.6 (50.2-530.2)**	
**ILD grade 1/2/3/4, n (%)**	**7 (41.1)/6 (35.3)/4 (23.5)/0 (0)**	

**ECOG PS Eastern Cooperative Oncology Group Performance Status, CEA carcinoembryonic antigen, CA19-9 cancer antigen 19 − 9, KL-6 Krebs von den Lungen-6 antigen, SP-D surfactant protein D**

### Selection and toxicity of subsequent chemotherapy after ILD

**Fig. 1:**
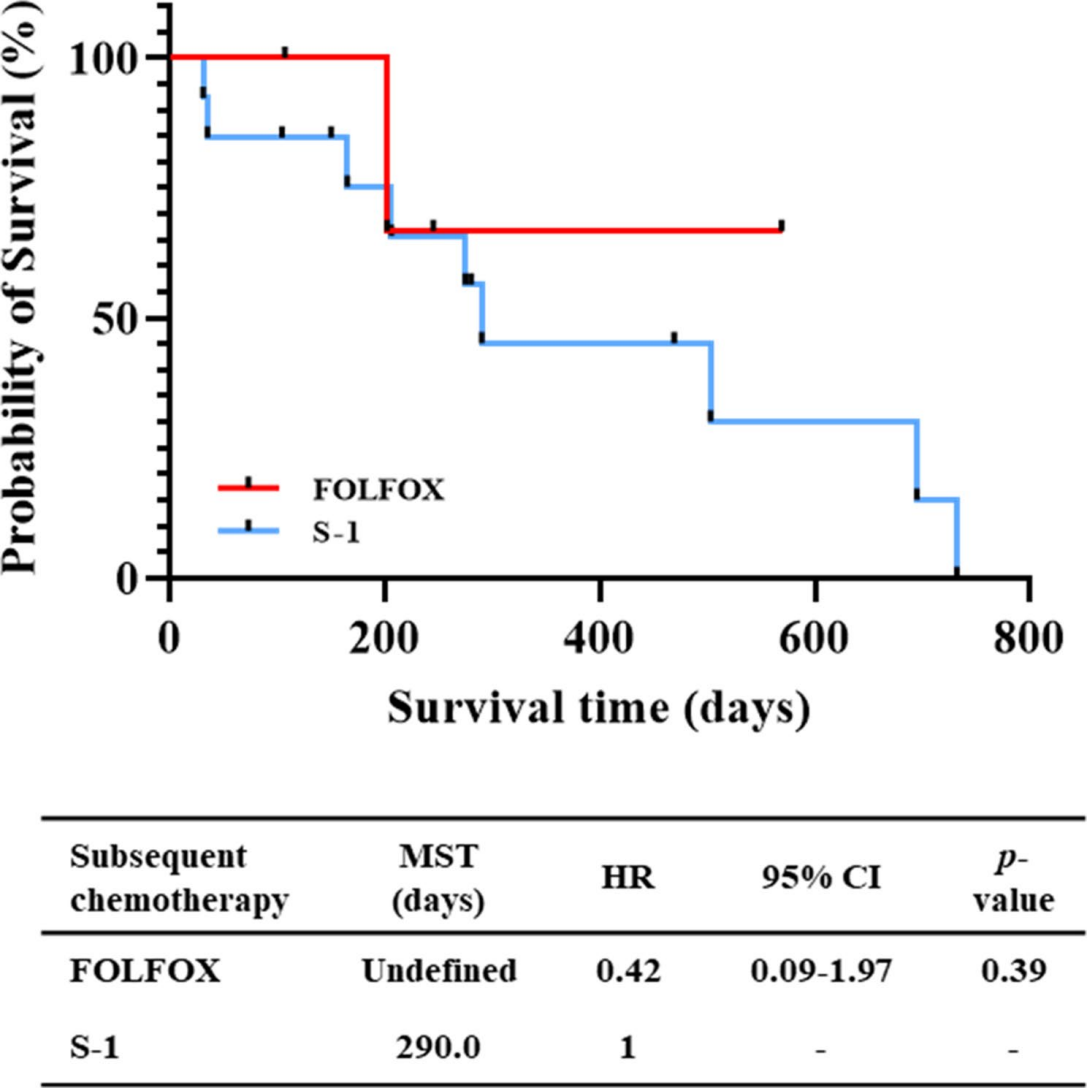
Survival analysis after the initiation of subsequent chemotherapy after recovery from ILD. ILD interstitial lung disease, MST median survival time, HR hazard ratio, CI confidential interval, chemo chemotherapy

We compared patient characteristics for each management strategy (S-1 monotherapy vs. FOLFOX) (Table 2). Among them, there was no significant difference in OS between the 2 regimens after the initiation of subsequent chemotherapy (FOLFOX vs. S-1: undefined vs. 290.0 days, p = 0.39) (Fig. 1). Regarding toxicity data for 17 patients who received chemotherapy, relapse of drug-induced ILD was not observed in all cases during the course. Overall, severe adverse events (CTCAE Grade 3 or 4) were observed in 3 patients (23.1%) in S-1 treatment group and 1 patient (25.0%) in FOLFOX treatment group. Regarding hematological adverse events, Grade 3 neutropenia was observed in 1 patient in both treatment group (7.6% vs. 25.0%, p = 0.34). Grade 3 non-hematological adverse events were observed only in S-1 treatment group (neuropathy and anorexia in 1 patient, respectively). A summary of CTCAE Grade 3 and 4 toxicities is given in Table 3.

**Table 2:** Comparison of patients’ characteristics

	S-1 n = 13	FOLFOX n = 4	p-value	
**Age (year-old)**	**70.5 (45–76)**	**67 (57–74)**	**0.61**	
**Sex (male:female)**	**11:2**	**2:2**	**0.21**	
**Tumor stage (cStage III: IV)**	**5:8**	**1:3**	**1.00**	
**ECOG PS (0:1)**	**13:0**	**4:0**	**1.00**	
**Smoking history, n (%)**	**7 (54.0)**	**2 (50.0)**	**1.00**	
**Brinkman index**	**960 (500–1530)**	**470 (200–740)**	**0.50**	
**History of lung disease, n (%)**	**0 (0)**	**0 (0)**	**1.00**	
**Presence of emphysema on CT scan, n (%)**	**3 (23.0)**	**1 (25.0)**	**1.00**	
**Treatment, 1st line, n (%)**	**11 (84.6)**	**4 (100.0)**	**1.00**	
**CEA (ng/mL)**	**6.6 (2.8–48.5)**	**2.6 (1.3–6.3)**	**0.06**	
**CA19-9 (U/mL)**	**73.9 (2.1–1990.0)**	**352.7 (130.4–4819)**	**0.11**	
**KL-6 (U/mL)**	**393.0 (187.0-1597.0)**	**271.5 (249.0-355.0)**	**0.13**	
**SP-D (ng/mL)**	**183.9 (88.0-442.4)**	**322.0 (145.7-530.2)**	**0.34**	
**ILD grade 1/2/3/4, n (%)**	**6 (46.2)/4 (30.8)/3 (23.1)/0**	**1 (25.0)/2 (50.0)/1 (25.0)/0**	**0.71**	
**Steroid therapy, n (%)**	**3 (23.0)**	**2 (50.0)**	**0.53**	

**ECOG PS Eastern Cooperative Oncology Group Performance Status, CEA carcinoembryonic antigen, CA19-9 cancer antigen 19 − 9, KL-6 Krebs von den Lungen-6 antigen, SP-D surfactant protein D**

**Table 3:** Grade 3 or 4 toxicity data according to the CTCAE (version 5.0)

	All cases n, (%)	S-1 n = 13	FOLFOX n = 4	p-value	
**Any grade ≥ 3 toxicity**	**4 (23.5)** **4**	**3 (23.1)**	**1 (25.0)**	**0.93**	
**Hematological toxicity grade ≥ 3**					
**Neutropenia**	**2 (28.5)**	**1 (7.6)**	**1 (25.0)**	**0.34**	
**Febrile neutropenia**	**0**	**0**	**0**		
**Thrombopenia/anemia**	**0**	**0**	**0**		
**Non-hematological toxicity grade ≥ 3**	**2 (28.5)**	**2 (15.3)**	**0 (0)**	**0.40**	
**Anorexia**	**1 (5.9)**	**1 (7.6)**	**0**	**0.56**	
**Neuropathy**	**1 (5.9)**	**1 (7.6)**	**0**	**0.56**	
**Nausea/Vomiting**	**0**	**0**	**0**		
**Diarrhea**	**0**	**0**	**0**		

**CTCAE the National Cancer Institute Common Terminology Criteria for Adverse Events**

## Discussion

This retrospective study aimed to clarify the optimal management of PDAC patients complicated with ILD induced by gemcitabine-based chemotherapy. Among the 17 enrolled patients, both S-1 monotherapy and FOLFOX showed comparable results in the survival analysis. Regarding toxicity data, no relapse of ILD was observed in the patients. Additionally, there were no significant differences in the prevalence of severe adverse events between the 2 groups. To the best of our knowledge, this is the first study to address the optimal management of PDAC patients complicated with ILD induced by gemcitabine-based chemotherapy.

Drug-induced ILD is not a rare adverse event in the current chemotherapy strategy for PDAC. Recently, Miyagahara et al. reported that 24 out of 390 patients (5.8%) developed ILD during chemotherapy in their multicenter study. The incidence of ILD in patients receiving gemcitabine-based chemotherapy has been reported to be significantly higher than that in patients administered non-gemcitabine-based chemotherapy regimens (22/452 vs. 2/236, p < 0.01) [15]. Additionally, the prognosis of PDAC patients with ILD has been reported to possibly be equivalent to that of patients without ILD if they are appropriately managed (11.5 vs. 11.4 months, p = 0.99) [12, 14]. Regarding following treatment after the onset of ILD, most of the patients in this study was treated with S-1 (71.4%) and no comparative study with other regimens could be performed. In our study, we found that 17 patients with subsequent treatment after the onset of ILD (S-1 in 13 patients and FOLFOX in 4 patients). In a comparative study for overall survival, we expected FOLFOX to have a superior effect to that of S-1 monotherapy [18, 20, 21]. However, FOLFOX failed to show superiority to S-1 monotherapy based on our results. Taking those findings into consideration, subsequent therapy with S-1 or FOLFOX can be an option for PDAC patients with ILD.

This study had some limitations. First, this study was a retrospective study with a small number of patients. Second, there might be selection bias between patients with and without subsequent treatment. While there were no statistically significant differences, the proportion of patients with poor PS was relatively high among patients without subsequent treatment. Additionally, the serum level of CA19-9 was markedly elevated in those patients. These facts implied that patients without subsequent treatment might have more advanced disease and poorer general health than those with subsequent treatment. To eliminate selection bias, propensity score matching may be an ideal statistical technique; however, recruitment of a sufficient number of patients is a difficult task due to the low prevalence of gemcitabine-based chemotherapy-induced ILD. Therefore, the results should be validated in a large nationwide study.

## Conclusion

Subsequent therapy with S-1 or FOLFOX can be an option for PDAC patients with ILD.

## Data Availability

The data that support the findings of the present study are available from the corresponding author upon reasonable request.

## Abbreviations

ILD: interstitial lung disease
PDAC: pancreatic ductal adenocarcinoma
OS: overall survival
GnP: gemcitabine plus nab-paclitaxel
BSA: body surface area
5-FU: 5-fluorouracil
PS: performance status
BI: Brinkman index
CT: computed tomography
CTCAE: Common Terminology Criteria for Adverse Events
CEA: carcinoembryonic antigen
CA19-9: cancer antigen 19 − 9
KL-6: Krebs von den Lungen-6 antigen
SP-D: surfactant protein D
